# Effect of the Protein Kinase B (PKB) Gene on the Carcinogenesis of Oral Squamous Cell Carcinoma in the South Indian Population

**DOI:** 10.7759/cureus.60099

**Published:** 2024-05-11

**Authors:** Devika Nair, Mahathi Neralla, Sushmaa Chandralekha Selvakumar, Auxzilia Preethi

**Affiliations:** 1 Oral and Maxillofacial Surgery, Saveetha Dental College and Hospitals, Saveetha Institute of Medical and Technical Sciences (SIMATS) Saveetha University, Chennai, IND; 2 RNA Biology Lab, Saveetha Dental College and Hospitals, Saveetha Institute of Medical and Technical Sciences (SIMATS) Saveetha University, Chennai, IND

**Keywords:** gene expression, apoptosis, proliferation, protein kinase b, oral squamous cell carcinoma

## Abstract

Introduction: The most common head and neck cancer is oral squamous cell carcinoma (OSCC). It is also one of the most prevalent forms of cancer globally. The current pharmacological treatment strategy for oral cancer lacks specificity and is capable of causing various side effects. This fact highlights the increasing need for targeted therapy. Interestingly, protein kinase B (PKB), commonly referred to as the AKT serine/threonine kinase, is an oncogenic protein that controls cell development, proliferation, apoptosis, and glycogen metabolism. Thus, the present study analyzed the AKT gene expression in OSCC patient samples.

Materials and methods: A total of 25 OSCC tissue samples and normal tissue samples were collected from the patients who reported to the Department of Oral and Maxillofacial Surgery, Saveetha Dental College and Hospitals in Chennai, India. The tissues were processed for H&E staining for histopathological confirmation, and expression studies of the AKT gene were done on both healthy and proven OSCC tissue samples. The data were shown as mean ± standard deviation, and p<0.05* was considered to be statistically significant.

Results: The quantitative reverse transcription polymerase chain reaction (qRT-PCR) analysis revealed that the AKT gene had been significantly upregulated in the OSCC tissue samples when compared to normal tissues (p<0.05). Moreover, upregulated AKT is postulated to be involved in increased cell proliferation and reduced apoptosis in OSCC.

Conclusion: The gene expression analysis was done in the samples of histologically confirmed OSCC, and it revealed that the AKT gene was significantly upregulated in OSCC tissues. Thus, AKT could be postulated as a potential therapeutic target for OSCC.

## Introduction

Head and neck cancers encompass a diverse group of malignancies occurring in various regions, including the oral cavity, pharynx, larynx, nasal cavity, and salivary glands. However, oral squamous cell carcinoma (OSCC) accounts for a significant majority of cases within this category. Oral squamous cell carcinoma has a significant global prevalence, making it one of the most commonly observed types of cancer. In India alone, it accounts for about 30% of all cancers and has an incidence of 20 per 100,000 cases [1]. Early diagnosis of oral cancer allows for effective treatment options, such as radiotherapy and surgery, to have curative effects. However, these treatments often come with adverse effects. In cases where the cancer has metastasized or if the individual has severe systemic side effects, the effectiveness of radiation and surgery is limited [2].

Conventional treatment methods, including surgery, radiation, and chemotherapy, may not always be suitable and can impact the prognosis and curative outcomes for oral cancer patients. Moreover, the current pharmacological treatment approach for oral cancer lacks specificity and can lead to various side effects. Given these challenges, there is a significant need for targeted therapy in the treatment of oral cancer. It is crucial to investigate and identify precise targets that can be effectively utilized for treating this type of cancer. Targeted therapy offers the potential for more specific and effective treatment, minimizing side effects, and improving the overall prognosis for patients with oral cancer [2, 3].

Interestingly, protein kinase B (PKB), commonly referred to as the AKT serine/threonine kinase, is an oncogenic protein that controls cell development, proliferation, apoptosis, and glycogen metabolism. Most human malignancies frequently exhibit overactivation of AKT [3]. Mutated or deleted PTEN is responsible for the overactivation of the phosphoinositol 3 kinase (PI3K)/AKT network, and restoration of the PTEN network inhibits PI3K/AKT signaling [3, 4]. The expression of specific oncogenes or the deletion of specific tumor suppressor genes can activate the PI3K/AKT signaling pathway [4]. Furthermore, we found that AKT was overexpressed in OSCC tissues compared to normal tissues. Activated AKT in terminal-stage OSCC tissues proves its importance in OSCC tumor growth and metastasis. Both AKT 1 and AKT 2 have also been found to increase the risk of other cancers like ovarian, prostate, pancreatic, and breast cancer [3]. Overexpression of the phosphorylated AKT protein has also been found to be present in 90% of oesophageal squamous cell carcinomas [4,5].

Activated AKT can regulate various essential signaling pathways involved in the carcinogenesis of oral cancer. Most of the genes involved in tumor growth, suppression, metastasis, and inflammation are associated with AKT. The downstream effectors of AKT include mammalian targets of rapamycin (mTOR), nuclear factor Kappa B (NF-κB), forkhead box O (FOXO), and so on. Deregulated mTOR signaling is linked to cancer, metabolic dysregulation, and aging [5]. The mTOR and PI3K/AKT signaling are essential for controlling many key cell activities, including protein synthesis, cell growth, metabolism, survival, catabolism, and autophagy. The expression of genes in cellular physiological processes such as apoptosis, cell-cycle control, glucose metabolism, and oxidative stress resistance is regulated by the FOXO family of transcription factors. AKT indirectly stimulates mTORC1 and directly phosphorylates FOXO to block it, which increases protein synthesis. NF-κB contributes to the regulation of inflammasomes and stimulates the expression of several pro-inflammatory genes, including those that produce cytokines and chemokines. Inhibition of NF-κB can lessen the pathological consequences by persistently activating the inflammatory response, which is possible with AKT inhibition [4,5].

The aim of this study is to analyze the expression levels of the AKT gene in OSCC tissues of the South Indian population and compare them with the expression levels in the normal tissues collected from the same patient, as well as to see if AKT can be postulated as a potential therapeutic target in the treatment of OSCC in the targeted gene pool.

## Materials and methods

Sample collection

The study was approved by the institutional ethics committee (approval number: IHEC/SDC/1981/22/OSURG/584), and all the samples were collected in accordance with the Helsinki Declaration. A total of 25 OSCC tissue samples and normal tissue samples were collected from the patients who reported to the Department of Oral and Maxillofacial Surgery, Saveetha Dental College and Hospitals, Chennai, India. The samples collected were stored in -200 C deep freezer storage for further analysis.

Inclusion and exclusion criteria

Participants had to be at least 18 years old, have the ability to give informed consent, and have histologically verified squamous cell carcinoma. Patients with squamous cell carcinoma of the buccal mucosa, oral tongue, floor of the mouth, maxillary and mandibular alveolus, hard palate, and gingivobuccal sulcus were included in the study.

Participants were eliminated from the study if they refused to participate in it. If the tissue sample size obtained was insufficient, it was rejected. Oropharyngeal squamous cell carcinoma (pharyngeal part of the tongue, soft palate, and other head and neck squamous cell carcinoma locations) was avoided.

Histopathological analysis

The tissues were processed for H&E staining for histopathological confirmation. The obtained tissue sections were grossed, processed, and embedded prior to sectioning. Sections were cut at 3 µm thickness, stained with H&E, and observed under a light microscope to reveal an invasive malignant epithelial neoplasm with malignant epithelial cells exhibiting features of severe epithelial dysplasia invading the connective tissue stroma as islands, strands, and cords with varying degrees of differentiation. 

Extraction and quantification of RNA

Total RNA was recovered from the tissues by homogenizing them in a homogenizer using TRIzol reagent (Invitrogen, Carlsbad, CA), as directed by the manufacturer. Thermo Fisher Scientific's Nanodrop 2000 Lite spectrophotometer (Thermo Fisher Scientific, Waltham, MA) was used to assess the RNA isolate's quality and quantity. The samples of RNA were kept at -20°C until they were examined further.

Reverse transcription

The process of reverse transcription involves combining the total RNA sample with oligo (dT) 18 primer (Promega, 50 μM), deoxyribonucleotide triphosphate (dNTP, 10 mM each) (New England Biolabs Inc., Ipswich, MA), and nuclease-free water. The mixture is then incubated at 65°C for five minutes, and the entire mixture is cooled immediately. Then, add 5x prime buffer (New England Biolabs Inc.), murine RNAse inhibitor (New England Biolabs Inc.), reverse transcriptase (New England Biolabs Inc.), and nuclease-free water to make up to 20 μl to this 10 μl template RNA primer mixture. The reaction mixture is incubated at 30°C for 10 minutes, 42°C for 30 minutes, 95°C for five minutes, and 40°C for the last incubation in a PCR (MiniAmp plus heat cycler, Thermo Fisher Scientific). The extracted cDNA is quantified in Nanodrop Lite and kept cold (-20°C) until additional examination.

Expression using quantitative reverse transcription polymerase chain reaction (qRT-PCR)

The generated cDNA is used in expression investigations for the gene AKT using Sybr Green (Takara Bio, Kutatsu, Japan). As a housekeeping control gene, GAPDH was employed. The expression investigations were conducted using the Bio-Rad CFX96 real-time system (Bio-Rad Laboratories, Inc., Hercules, CA). The initial denaturation at 95°C for 30 seconds for one cycle, the denaturation at 95°C for five seconds, and the annealing for 30 seconds up to 40 cycles with a melt curve constituted the PCR cycling temperature. The experiments were run in duplicate, and the gene expression was determined using the 2^-∆∆Cq technique. Table 1 represents the primers used in the experiment. The data presented are the mean of duplicate experiments+standard error of the mean (SEM). The statistical difference between the groups was calculated using the Student's t-test in Microsoft Excel 365 software (Microsoft Corp., Redmond, WA). A p-value <0.05 was considered statistically significant (*).

**Table 1:** Primers used in the experiment

Primers	Forward sequence	Reverse sequence
GAPDH	F-GTCTCCTCTGACTTCAACAGCG	R-ACCACCCTGTTGCTGTAGCCAA
AKT	F– TTCTGCAGCTATGCGCAATGTG	R– TGGCCAGCATACCATAGTGAGGTT

## Results

Clinical characterization of participants

Only 16 individuals were selected as study participants, consisting of 13 men and three women with a mean age ranging from 35 to 60 years. Eleven individuals chewed pan and gutka, six were smokers, and eight were drinkers. According to tumor staging, nine participants had T1 and T2 stage tumors, while seven had T3 and T4 stage tumors. The histological grades of well- and moderately differentiated squamous cell carcinoma were eight each. Interestingly, the study showed four non-habit cases who had OSCC. Table 2 displays the clinical information about the individuals.

**Table 2:** Clinical characteristics of the participants

Clinical features		Total cases (n=16)
Age (years)	<50	11
	>50	5
Gender	Male	13
	Female	3
Tobacco chewing habit	Yes	12
	No	4
Alcohol habit	Yes	12
	No	4
Histological grade	Well-differentiated	8
	Moderately differentiated	8
Tumor staging	T1-T2	9
	T3-T4	7
Tissue subsite	Right buccal mucosa	8
	Left buccal mucosa	8

Histopathological analysis of OSCC and normal tissue

**Figure 1:**
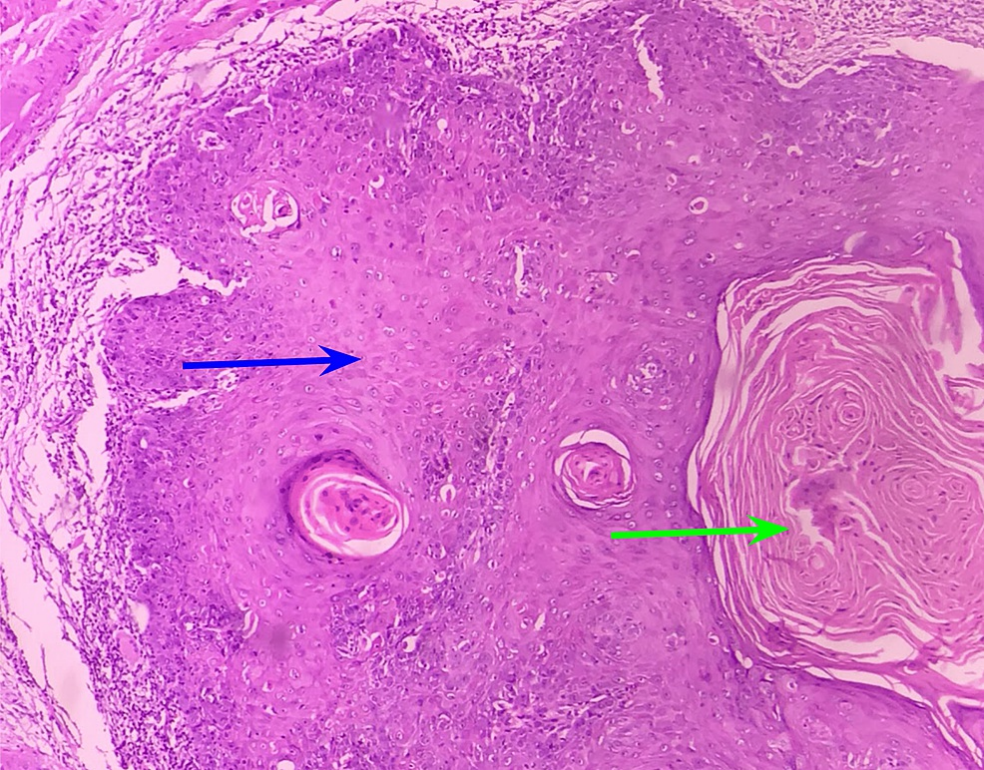
This figure represents the H&E staining of OSCC sections. The blue arrow indicates the tumor cells and the green arrow indicates the keratin pearls. OSCC: oral squamous cell carcinoma

Malignant epithelial cells were seen in OSCC tissue samples as islands, cords, or strands infiltrating the connective tissue stroma and exhibiting severe epithelial dysplasia characteristics such as increased nucleocytoplasmic ratio, dyskeratosis, and mitotic figures. Keratin pearls were also observed in well-delineated OSCC, as seen in Figure 1.

The OSCC instances included both well-differentiated and moderately differentiated OSCCs. Normal tissue revealed parakeratinized stratified squamous epithelium devoid of epithelial dysplasia. 

Expression analysis of the AKT gene in OSCC vs. normal tissues

**Figure 2:**
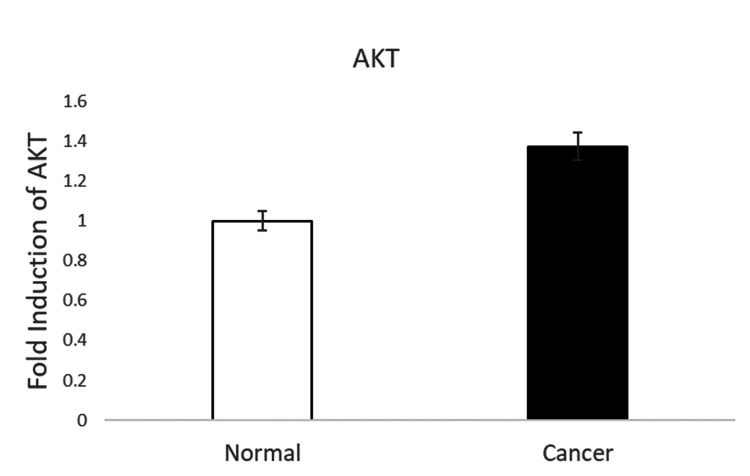
This figure depicts the expression analysis of the AKT gene in OSCC and normal tissues (*p<0.05) The values presented here are in percentage, mean +/- SD, p<0.05. OSCC: oral squamous cell carcinoma

The qRT-PCR analysis revealed that the gene AKT had been significantly upregulated in the OSCC tissue samples when compared to normal tissues. Moreover, upregulated AKT is postulated to be involved in increased cell proliferation and reduced apoptosis in OSCC. Figure 2 depicts the expression analysis of the AKT gene in OSCC and normal tissues.

## Discussion

The AKT gene activates tumor growth factors and suppresses tumor-suppressing genes. Since the AKT gene is an essential regulator of tumorigenesis, inhibiting the activation of the AKT gene could be a potential therapeutic target for oral cancer. Thus, AKT inhibition could be made possible by identifying molecules that target AKT. Finding AKT inhibitors that can directly prevent PI3K/AKT signaling by obstructing pAKT expression of AKT kinase activity could slow cancer development. Various synthetic, as well as natural compounds, serve as AKT inhibitors [6]. The invasion and metastasis of cancers depend heavily on the migration and adhesion of cancer cells. Cancer cells had trouble moving when AKT was inhibited because it significantly decreased AKT phosphorylation or decreased the expression of AKT downstream proteins [7].

In recent years, microRNAs (miRNAs or miR) have been studied for their potential role in regulating various genes in disease progression and regression. Their role in cancer therapeutics is also widely studied [2]. Various miRNAs regulate AKT in oral cancer. For instance, in one of the studies by Iizumi et al. (2021), miR-142-5p was found to regulate PI3K/AKT signaling in OSCC [8]. It was identified that miR-142-5p targets the phosphatase and tensin homologue (PTEN) gene and contributes to cancer development. PTEN is one of the tumor suppressor genes that play a vital role in inhibiting the PI3K/AKT signaling pathway. The study implied that miR-142-5p controls the PI3K/AKT pathway by targeting the PTEN gene, contributing to the development of OSCC. Thus, it could be understood that inducing PTEN could downregulate AKT activation and its associated downstream pathways [9]. Another study has discussed the role of miR-16 in inhibiting AKT in OSCC. It was identified that miR-16 mimics decreased AKT3 and BCL2L2 expression, reducing tumor weights and volumes, but miR-16 inhibitors had adverse effects. Thus, the results concluded that miR-16 acts as a tumor suppressor miRNA to reduce the oncogenes AKT3 and BCL2L2 in OSCC, hence inhibiting cell growth and inducing apoptosis [10]. 

In a study by Li et al. [11], increased AKT expression was observed in human ameloblastoma tissues. It was also identified that phosphorylated AKT was involved in the downstream activation of mTOR and led to cancer progression. Some matrix metalloproteinases, including MMP-2, may have their expression upregulated by AKT in a PI3K/AKT/mTOR-dependent manner. The AKT gene may have an impact on cell movement, invasiveness, and the morphological properties of epithelial cells. To increase the movement of cancer cells on the cellulose membrane, activated AKT may promote epithelial-mesenchymal transition (EMT) by down-regulating E-cadherin and beta (β)-catenin expression and up-regulating mesenchymal vimentin expression [12-14]. The AKT gene activates lipid and protein kinase, which sets off a series of reactions that propel the growth and proliferation of cells as well as their survival and motility, ultimately leading to the formation of tumors [15]. AKT is not only involved in tumor growth but also chemoresistance. Drug resistance is a common incidence of cancer, and AKT is found to be one of the critical regulators of drug resistance in cancer [11]. The PKB gene's ability to activate a number of pro-apoptotic proteins via stimulating cell survival signals via the PI3K pathway has drawn attention to the protein [16]. This protein is a potential new target for molecular therapeutics, acting also as a target for drug discovery as it has ectopic expression in a number of other malignancies [17-22]. Studying the expression levels of the AKT gene helps in molecular understanding, biomarker identification, therapeutic targeting, and the identification of potential synergistic treatment combinations. Our study is the first of its kind, targeting the South Indian population. There aren't many studies with a sole focus on the South Indian gene pool.

Oral squamous cell carcinoma is a heterogeneous disease with diverse genetic and molecular characteristics. The expression of AKT can vary among different tumor samples, making it challenging to draw definitive conclusions from gene expression studies. The number of patient samples included in a study can impact the statistical power and reliability of the gene expression results. Small sample sizes may lead to limited generalizability and increase the risk of false-positive results. AKT is part of a complex network of signaling pathways, and its gene expression alone may not capture the full complexity of its functional activity. Therefore, solely examining AKT gene expression may not fully help in understanding its functional status in OSCC. Gene expression studies provide valuable insights into the expression levels of specific genes; they do not directly assess the functional activity or post-translational modifications of AKT. Additional assays, like protein expression and pathway-specific functional studies, may be necessary to validate and correlate the gene expression of AKT.

## Conclusions

To conclude, since AKT is found to be an important oncogene in the tumor growth and metastasis of various cancers, including OSCC, its inhibition is postulated to be a potential treatment against OSCC. This could be made possible with the help of molecules like miRNAs that regulate AKT. Thus, exploring the role of miRNAs and AKT could solve the mysteries of the molecular mechanisms and signaling pathways involved in OSCC. Longitudinal studies may help in tracking changes in AKT gene expression over time and response to treatments, which would provide more comprehensive insights into its role in OSCC progression and response to therapy. The study of AKT gene expression in OSCC contributes to our understanding of the disease and may have implications for the development of targeted therapies. Future research should aim to overcome these limitations and integrate multiple approaches to gain a comprehensive understanding of the role of AKT in OSCC.
